# Benchmarking foundation cell models for post-perturbation RNA-seq prediction

**DOI:** 10.1186/s12864-025-11600-2

**Published:** 2025-04-23

**Authors:** Gerold Csendes, Gema Sanz, Kristóf Z. Szalay, Bence Szalai

**Affiliations:** Turbine Ltd., Budapest, Hungary

**Keywords:** Perturbation, Foundaton model, RNA-seq, Benchmark

## Abstract

Accurately predicting cellular responses to perturbations is essential for understanding cell behaviour in both healthy and diseased states. While perturbation data is ideal for building such predictive models, its availability is considerably lower than baseline (non-perturbed) cellular data. To address this limitation, several foundation cell models have been developed using large-scale single-cell gene expression data. These models are fine-tuned after pre-training for specific tasks, such as predicting post-perturbation gene expression profiles, and are considered state-of-the-art for these problems. However, proper benchmarking of these models remains an unsolved challenge.

In this study, we benchmarked two recently published foundation models, scGPT and scFoundation, against baseline models. Surprisingly, we found that even the simplest baseline model—taking the mean of training examples—outperformed scGPT and scFoundation. Furthermore, basic machine learning models that incorporate biologically meaningful features outperformed scGPT by a large margin. Additionally, we identified that the current Perturb-Seq benchmark datasets exhibit low perturbation-specific variance, making them suboptimal for evaluating such models.

Our results highlight important limitations in current benchmarking approaches and provide insights into more effectively evaluating post-perturbation gene expression prediction models.

**Supplementary Information:**

The online version contains supplementary material available at 10.1186/s12864-025-11600-2.

## Introduction

Modelling cellular phenotypes is a fundamental challenge in computational systems biology. Accurately predicting cell fate can advance our understanding of both healthy and diseased states and facilitate the identification of novel therapeutic targets. Over the past few decades, computational models based on Boolean logic [1] and ordinary differential equations (ODEs) have been developed [2]. More recently, advances in deep learning methodologies have reinvigorated interest in this area [3–8].

Cellular phenotypes can be described using various data modalities, including transcriptomics, (phospho)proteomics, and imaging-based phenotypic assays (phenomics). Among these, transcriptomics data —derived from techniques such as microarrays, bulk RNA sequencing, and single-cell RNA sequencing (scRNA-seq)— is the most used for large-scale cellular phenotype analysis due to its relatively low cost and well-established analysis methods [9]. While gene expression profiles do not directly reflect protein-mediated signalling, they offer a proxy for the overall cellular state [10]. Notably, post-perturbation transcriptomics data are particularly suited for training computational models because the causal relationship between known perturbations (cause) and the measured post-perturbation gene expression (effect) allows for the modelling of mechanistic processes [11]. However, acquiring such perturbation data is more complex than obtaining baseline (non-perturbed) transcriptomics data.

To address these challenges, several Transformer-based, foundation cell models have emerged recently [4, 6, 7]. These models are pre-trained on vast amounts (> 10 M examples) of unlabelled scRNA-seq data, with the aim that such large datasets allow the models to capture general principles of gene regulation and signalling. These pre-trained models can then be fine-tuned on perturbation data to predict post-perturbation phenotypes more effectively.

These models had shown strong performance in post-perturbation RNA-seq prediction tasks using Perturb-seq-based genetic perturbation benchmarks [12]. These models generally predict the post-perturbation RNA-seq profiles of perturbed single cells by using gene expression data from unperturbed cells, along with a representation of the perturbation. The primary evaluation for these models is their ability to predict RNA-seq profiles for unseen perturbations.

However, benchmarking machine learning models in this domain is challenging. We have previously demonstrated that test set selection and performance metrics can significantly impact benchmarking outcomes in the related problem of post-perturbation viability prediction [13]. Poor test set design or inappropriate metric choice can result in indistinguishable performance between well-performing and trivial models. Furthermore, computational models of cellular phenotypes have diverse applications, such as predicting the effects of known perturbations in novel cell types (Cell Exclusive, CEX setup) or predicting the effects of novel perturbations in familiar cell types (Perturbation Exclusive, PEX setup). Unfortunately, current benchmarks, which predominantly rely on Perturb-seq datasets comprising diverse genetic perturbations in a single cell line, primarily assess PEX performance, limiting their ability to evaluate generalisation in a broader context.

In this study, we benchmarked two recent transformer-based foundation models, scGPT and scFoundation, across four Perturb-seq datasets. We compared their performances to baseline models of varying complexity. Surprisingly, we found that foundation models generally underperformed compared to a simple baseline model that uses the mean of the training samples. Standard machine learning models using biological prior-knowledge outperformed foundation models by a large margin. We further identified that the low inter-sample variance in commonly used datasets complicates model performance assessment.

## Results

### Benchmarking of post-perturbation RNA-seq prediction methods

scGPT and scFoundation are large language model (LLM) based transformer architectures, pre-trained on large-scale, unlabelled single-cell RNA sequencing (scRNA-seq) data. Through pre-training, scGPT and scFoundation learn gene embeddings and capture gene–gene relationships, which can then be leveraged for various downstream tasks, including post-perturbation RNA-seq prediction. The models take as input RNA-seq vectors from randomly selected unperturbed cells, along with a perturbation representation, to predict RNA-seq profiles of perturbed cells. scGPT uses a perturbation token, which is added to the perturbed gene token to model perturbation effects, while scFoundation uses the pretrained gene embeddings as inputs for the graph neural-network based GEARS [5] model for post-perturbation RNA-seq prediction. For both models, we used the pretrained models from the original publications, and we fine-tuned them on the benchmark datasets according to the authors description.

For benchmarking, we employed the three datasets from the scGPT paper and extended it with another perturbation RNA-seq dataset. These datasets were generated using Perturb-seq, which combines CRISPR-based perturbations with single-cell sequencing to capture post-perturbation gene expression profiles. Specifically, the Adamson [14] dataset comprises 68,603 single cells subjected to single perturbation CRISPR interference (CRISPRi). The Norman dataset [15] includes 91,205 single cells subjected to single or dual CRISPRa (overexpression). Lastly, two subsets [5] of the Replogle [16] dataset containing 162,751 and 162,733 single cells (K562 and RPE1 cell lines, respectively) from a genome-wide single perturbation CRISPRi screen were used. We evaluated the Perturbation Exclusive performance of models in our study—assessing their ability to handle unseen perturbations or, in the case of the Norman dataset, unseen combinatorial perturbations (Supplementary Table 1).

**Fig. 1:**
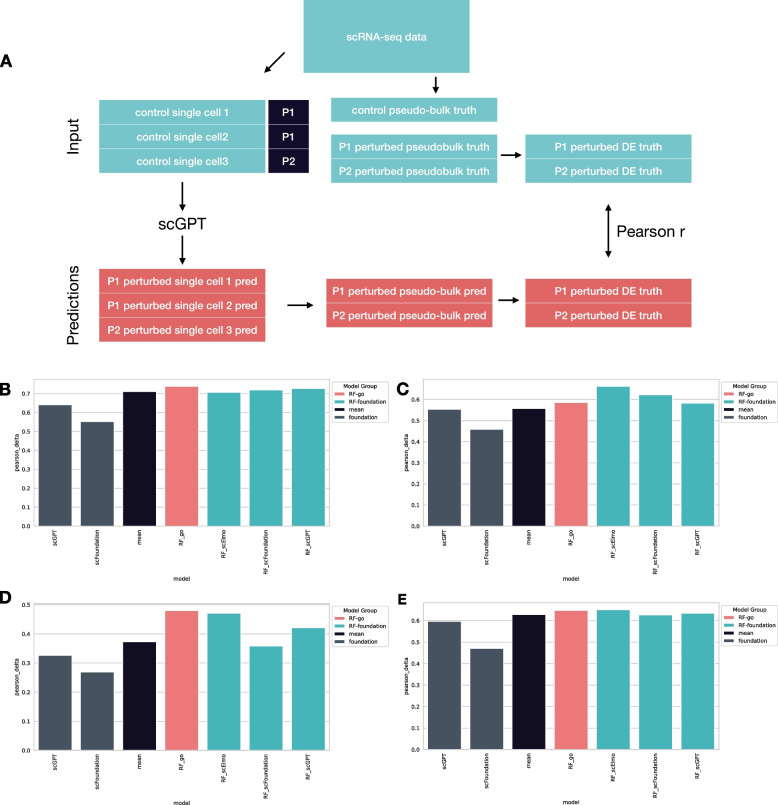
Benchmarking foundation and baseline models (**A**) Schematic representation of benchmark pipeline (**B**) Evaluation on the Adamson dataset: Pearson delta metrics (y axis) for scGPT, scFoundation, Train Mean and Random Forest Regression model with different features (x axis). Main groups of models a colour coded. **C** Evaluation on the Norman dataset (**D**) Evaluation on the Replogle K562 dataset (**E**) Evaluation on the Replogle RPE1 dataset

For evaluation, we adopted the metrics used by the scGPT authors (Fig. 1A). Predictions were generated at the single-cell level, and the predicted gene expression profiles for each perturbation were averaged to form pseudo-bulk expression profiles. These predicted profiles were then compared to the ground truth pseudo-bulk profiles using Pearson correlation coefficients. Importantly, Pearson correlations were calculated not only in the raw gene expression space but also in the differential expression space (i.e., perturbed gene expression profile minus control gene expression profile). Additionally, we evaluated performance on the top 20 differentially expressed (DE) genes to emphasise the model's ability to capture the most significant transcriptional changes. To identify DE genes, we used a t-test based approach corresponding to the scGPT publication, and also a Wilcoxon test-based approach (Methods).

To further assess prediction performance, we introduced several baseline models. The simplest model, Train Mean, predicted post-perturbation expression by averaging the pseudo-bulk expression profiles from the training dataset. Consequently, all predicted gene expression vectors are identical and set to this average vector. We also constructed Elastic-Net Regression (EN), k-Nearest-Neighbors (kNN) Regression and Random Forest Regressor (RF) models. These models took as input different prior knowledge-based features of the perturbed genes, namely Gene Ontology (GO) vectors [17], embeddings from scGPT, scFoundation and scELMO [18]. scELMO is a method which creates embeddings of genes, using a text description of them that is generated by LLMs like GPT 3.5. For the baseline models, we used pseudo-bulked expression profiles on the target side (detailed in Methods).

Our results show that the scGPT models we reproduced achieved similar performance to those in the original publication (Fig. 1B-E; Supplementary Table 2). In the raw gene expression space, all models performed similarly (Pearson > 0.95, Supplementary Table 2). However, the Pearson correlation values between raw gene expression profiles are strongly influenced by the baseline expression magnitudes of different genes, so we did not consider these metrics meaningful.

In the differential expression space (Pearson Delta, Fig. 1B-E; Supplementary Table 2), even the simplest baseline model, Train Mean reached better correlation (0.711, 0.557, 0.373 and 0.628 for Adamson, Norman, Replogle K562 and Replogle RPE1, respectively) than foundation models (scGPT: 0.641, 0.554, 0.327 and 0.596, scFoundation: 0.552, 0.459, 0.269 and 0.471 for the four datasets, respectively). Random Forest Regressor with GO features outperformed foundation models by a large margin (0.739, 0.586, 0.480 and 0.648, for the four datasets, respectively). In the Norman dataset, where combinatory perturbations were used, we also analysed the model performance for different subgroups: where 0, 1 or 2 perturbation of the combination was present in the train test, and for new single perturbations. Train Mean and RF with GO features also outperformed foundation models in this subgroup analysis (Supplementary Fig. 1 and Supplementary Table 3). Generally, EN and kNN models had better performance than foundation ones, but weaker than RF models (Supplementary Table 2).

As RF model with GO features outperformed foundation models, we were interested whether this low performance of foundation models is a consequence of their general inability to learn biological meaningful representation (i.e., embeddings) of perturbations. To test this, we used the pretrained foundation model embeddings as features of the RF model (Fig. 1B-E, Supplementary Table 1). We found that RF model using the pretrained embeddings had better performance, than the finetuned models itself, especially in case of scGPT (Pearson Delta metrics: 0.727, 0.583, 0.421 and 0.635 for Adamson, Norman, Replogle K562 and Replogle RPE1, respectively), however still had weaker performance than RF with GO features. We also explored the performance of recent natural language processing-based gene embeddings from scELMO. RF models using scELMO features had similar performance (0.706, 0.663, 0.471 and 0.651, for the four datasets respectively) as GO based RF models. To further analyse the differences between embeddings, we compared the mean similarity (Pearson correlation between embedding vectors, Methods) of gene embeddings sharing biological pathways (KEGG [19], REACTOME [20]) or gene regulatory networks (CollecTRI [21]). We found that GO feature similarity had the highest intra-pathway similarity, followed by scELMO and scGPT, except in case of the CollecTRI gene regulatory network database, where scGPT outperformed scELMO (Supplementary Fig. 2).

We also analysed the model performance at the level of the top 20 differentially expressed (DE) genes, according to the original scGPT publication. Using the correlation between the top 20 differentially expressed genes (Pearson Delta DE, Supplementary Table 1), scGPT had better performance than Train Mean. However, in the Top20 DE genes the CRISPR target gene of the perturbation was frequently present. Since the target genes’ expression significantly decreased in CRISPRi experiments (Adamson, Replogle) and increased in the CRISPRa experiment (Norman), and scGPT used a perturbation token directly associated with gene tokens, predicting target gene expression can be considered trivial. Removing the target genes from the Top20 DE gene list resulted in a decrease in scGPT's performance in the Top20 DE space (Supplementary Table 1). Using Wilcoxon test instead of t-test for DE calculation did not affect these results (Supplementary Table 2).

In summary we found that even the simplest predictor, the Train Mean model achieved better performance than foundation models regarding predicting the post-treatment RNA-seq vectors of perturbed cells, and the simple Random Forest Regressor model outperformed foundation models by a large margin.

### Limited perturbation diversity biases benchmarking outcomes

**Fig. 2:**
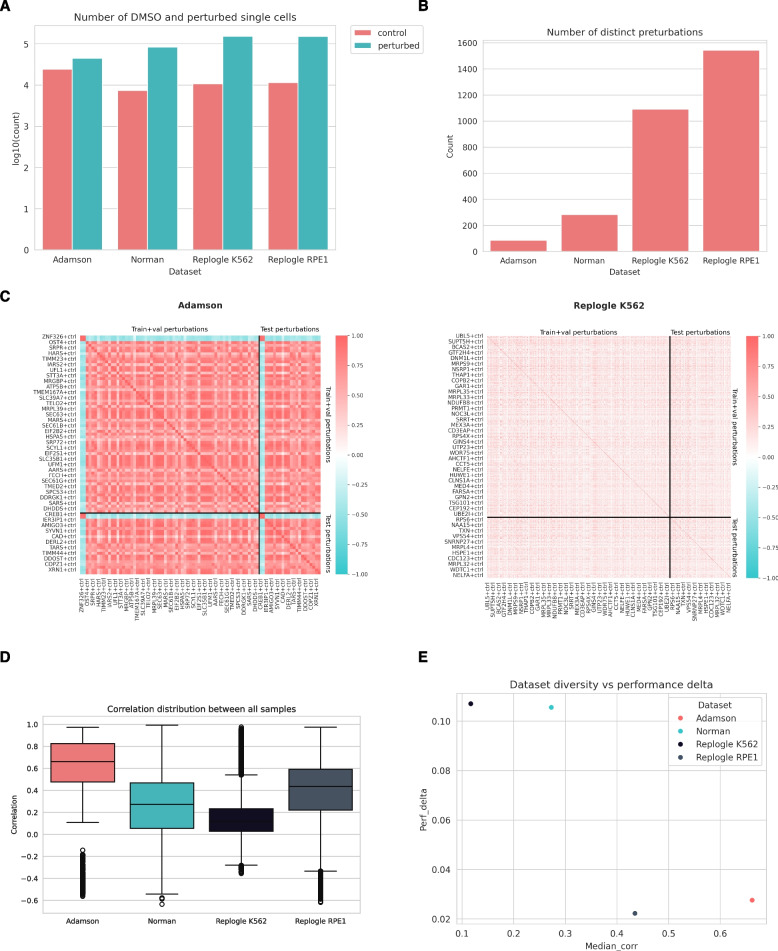
Composition of standard benchmark datasets (**A**) Number of single cells (control/perturbed, colour code) in the four benchmark datasets. y axis is log10 scaled (**B**) Number of distinct perturbations in the four benchmark datasets (**C**) Correlation heatmaps for pseudo-bulk differential expression signatures for Adamson (left), and Replogle K562 (right) datasets. The black lines indicate the separation between training and test sets. Some samples (perturbations) are labelled on the x and y axes. **D** Distribution of pairwise Pearson correlations (y axis) of differential expression profiles for the benchmark datasets (x axis). **E** Comparison between median intra-dataset correlations (x axis) and Pearson Delta metrics difference between best and Train Mean models (y axis), colour coded by the benchmark dataset

To explore the unexpected strong performance of the simple Train Mean model, we examined the composition of the benchmark datasets in more detail (Fig. 2A). Although the four Perturb-seq datasets contain a large number of single cells (ranging from ~ 70,000 in the Adamson dataset to ~ 160,000 in the Replogle datasets), the number of distinct perturbations is comparatively much smaller —87 in Adamson, 284 in Norman, 1,093 in Replogle K562 and 1,544 in the Replogle RPE1 (Fig. 2B). While having multiple single cells per perturbation can be beneficial for model training (i.e., fine-tuning) as it provides insights into perturbation-specific gene expression variability, it would be surprising that large-scale models could efficiently learn and generalise from such a limited number of distinct perturbations.

To further investigate the characteristics of the benchmark datasets, we analysed the pairwise similarities between pseudo-bulk differential expression profiles—computed as the difference between perturbed and control log-transformed gene expression vectors. Specifically, we calculated Pearson correlations between these differential profiles for all perturbation pairs within each dataset (Fig. 2C, Supplementary Fig. 3). We reasoned that the distribution of these correlation values (Fig. 2D) reflects the heterogeneity of a dataset: a high median correlation indicates that perturbations elicit similar transcriptional responses (i.e., low heterogeneity), while a lower median correlation suggests more diverse responses across perturbations (i.e., high heterogeneity). In the Adamson dataset, we observed high similarity between the perturbation profiles, with a median Pearson correlation of 0.662, suggesting a low heterogeneity in the Adamson dataset. This result is not entirely unexpected, given that the Adamson study focused on perturbations specifically targeting endoplasmic reticulum homeostasis, where similar transcriptional responses might be expected. Only a few genes exhibited anti-correlated expression profiles. In contrast, the Replogle K562 dataset displayed greater variability in the perturbation profiles, with a lower median Pearson correlation of 0.117. This greater variability aligns with the genome-wide scope of the Replogle study, where perturbations target a broader range of biological pathways, leading to more diverse transcriptional outcomes.

To analyse the effect of dataset heterogeneity on benchmarking, we plotted the above-described intra-dataset median perturbation correlations against the performance difference between the best model and Train Mean for each benchmark dataset (Pearson Delta values, Fig. 2E) – this later metric represents the dynamic range of benchmark dataset for identifying good performing models. We found an inverse relationship between intra-dataset median correlation and delta model performance, suggesting that more diverse datasets (Norman, Replogle K562) are better suited to identify good performing models.

In summary, our analysis indicates that while these benchmark datasets contain many single cells, the actual biological variance between the samples is relatively small. This limited variance likely explains why simple models, such as averaging the training examples, perform unexpectedly well in terms of evaluation metrics. However, this small variance also hampers the benchmarks' ability to effectively differentiate between more complex models, thereby limiting the assessment of their true performance.

## Discussion

In this study, we compared the post-perturbation RNA-seq prediction performance of foundation models against several baseline models using Perturb-seq datasets. Surprisingly, we found that even the simplest baseline, the Train Mean model, outperformed scGPT and scFoundation in most cases. A basic machine learning model, Random Forest Regressor, which incorporated biological prior knowledge in the form of Gene Ontology (GO) terms, outperformed foundation models by a large margin. These results align with a preprint published during the preparation of our manuscript [22]. Our findings also suggest that the current benchmark datasets, particularly the Adamson and Replogle RPE studies, may lack sufficient variability to accurately distinguish between the performances of different models.

Given that these benchmark datasets are primarily designed to evaluate the PEX problem —predicting responses to novel perturbations— the correct representation of perturbations is essential for model performance. While scGPT and scFoundation are pre-trained on large-scale, unlabelled single-cell RNA-seq data and likely learns gene regulatory interactions, it does not utilise perturbation data during pre-training. Our comparison of embedding similarities also demonstrated, that scGPT embeddings show smaller associations with pathway information (KEGG, REACTOME) than Gene Ontology features, or natural language processing based (scELMO) embeddings. Interestingly, scGPT embeddings have stronger association with a gene regulatory network (CollecTRI) than scELMO, also underlying that current foundation models trained on unlabelled scRNA-seq data are learning gene regulatory, but not protein interaction networks. Perturbation-specific information must be learned during fine-tuning of the foundation models. The relatively small number of distinct perturbations in the benchmark datasets may not be sufficient to capture this complexity. In contrast, the Random Forest Regressor’s use of GO terms as prior knowledge for perturbation representation appears to provide a more effective means of modelling perturbation responses. This suggests that perturbation effects, which propagate through signalling networks, may be better represented by functional categories such as GO terms than by the gene regulatory networks primarily learned by foundation models.

Our analysis also highlights a significant limitation in the current benchmark datasets—low variance. The Adamson and Replogle RPE1 datasets exhibit high similarity between perturbations, which hinders the ability to distinguish between model performances. Additionally, the current benchmarks are focused exclusively on the PEX problem, neglecting the CEX problem, which involves predicting responses in novel cell types. Datasets such as LINCS-L1000 [23], which feature a larger number of cell lines and perturbations, or Mix-Seq [24], could serve as valuable resources for addressing both PEX and CEX setups and improving future benchmarks. However, these datasets are either hybridization based (LINCS-L1000) or are utilizing drug perturbations, thus incorporating them into existing benchmarks requires further development.

Our findings also raise questions about the utility of single-cell RNA-seq data for post-perturbation RNA-seq prediction. While single-cell data allow for the modelling of cellular heterogeneity and provide larger sample sizes, the baseline models we used operated on pseudo-bulked data and performed comparably or better than foundation models. Single-cell expression profiles are often subject to technical noise, such as dropout events, which may obscure meaningful biological signals. In our current benchmark study, using single-cell data did not provide an obvious advantage over pseudo-bulked data, particularly given that the datasets were derived from in vitro cell lines with low heterogeneity.

Recently, several other benchmarks have been suggested for perturbation RNA-seq predictions [22, 25, 26]. The most important differences of our benchmarking are the following: 1) We show that Pearson correlation in the raw gene expression space is not a useful metric for comparing model performances, instead correlation of differential expression profiles (Pearson Delta) should be used. This metric has been previously shown to be useful to compare gene expression signature similarities [23, 27], thus the similarity between predicted and observed gene expression profiles. 2) We analysed in detail the usability of different features (foundation model embeddings, scELMO, GO) for perturbation RNA-seq prediction. 3) We also highlight the how dataset diversity influences the benchmarking process.

In summary, our benchmarking revealed that foundation models performed comparably to the trivial Train Mean model in post-perturbation RNA-seq prediction tasks and were outperformed by a Random Forest Regressor utilising prior biological knowledge. Furthermore, the low variance in the commonly used benchmark datasets limits their ability to effectively assess model performance. Although our analysis focused on scGPT and scFoundation, other foundational models that have been tested on the same datasets may face similar limitations. While single cell foundational models like scGPT hold promise due to their ability to incorporate large-scale, unlabelled data, several recent studies [28, 29] suggest that their performance on certain tasks may lag behind state-of-the-art or even baseline models. Moving forward, more rigorous and meaningful benchmarks that include higher variance and incorporate diverse datasets are needed to properly assess the applicability of machine learning models in post-perturbation prediction tasks.

## Methods

### Benchmark datasets

The benchmark datasets (Adamson [14], Norman and two Replogle [16] datasets) were downloaded and processed by the GEARS’ cell-gears v0.0.1 package [5]. Single cell expression values were normalised to 10000 reads and log transformed. Top 20 mostly differentially expressed genes were used from the original GEARS publication to ensure the consistency of our benchmark with the published results. The top 20 DE genes were calculated with the *tl.rank_genes_groups()* function of Scanpy [30] with default parameters. To ensure that our benchmark is not affected by DE calculation, we also selected the top 20 DE genes using Wilcoxon test (*tl.rank_genes_groups(method* = *’wilcoxon’)*)*.* All benchmark datasets were split into train, validation and test sets according to the GEARS publication - corresponding to Perturbation Exclusive split, where new, unseen perturbations (or perturbation combinations) were present in the test set (Supplementary Table 1).

### Foundation models

We reproduced scGPT’s results on the GEARS benchmarking datasets using the cell-gears v0.0.1 package and a fork of scGPT v0.2.1 (commit 7301b51). The default hyperparameters were kept, most notably; model and feedforward dimensions at 512, number of layers at 12, number of heads at 8. Fine tuning was conducted for 15 epochs, selecting the best-performing epoch based on validation set performance. All training runs were executed on Nvidia A100 GPUs (80 GB). The fine-tuning procedure was applied consistently across all four datasets: Adamson, Norman, Replogle K562, and Replogle RPE1. To train the scFoundation model on the benchmark datasets, its corresponding repository at the commit 69b0710 was used. ScFoundation was fine tuned for 10 epochs with an effective batch size of 32 on all tasks. By default, scFoundation trains for 15 epochs, using an effective batch size of 30. By epoch 10, the model already converged and thus, an early stopping was sensible in all tasks. The rest of the hyperparameters were kept constant. The model was trained on the same hardware as scGPT.

### Baseline models

scGPT’s performance was compared against four baseline models: 1) Train Mean 2) Random Forest Regressor, 3) Elastic Net and 4) k-Nearest-Neighbour (kNN) Regressor. The Train Mean model predicts the same vector for each test sample, calculated as the mean post-perturbation pseudo-bulk expression across all training samples for each perturbation. Other baseline models used different prior knowledge-based embeddings of the perturbed gene as features, and the pseudo-bulked gene expression profiles (same genes as for foundation models) as targets. We used the same splits for foundation and baseline models. Gene Ontology terms for all genes were downloaded using the decoupler Python package [31], and reformatted as a binary gene – Gene Ontology term indication matrix. PCA dimensionality reduction was applied to the GO matrix (n_genes, n_functions), reducing it to 256 principal components. For each sample, the perturbed gene was identified from the PCA-transformed GO features. In cases where multiple perturbations occurred in a single sample, the embeddings were simply summed. When using scElmo’s, scGPT’s, scFoundations embeddings as an alternative to the GO, the same preprocessing was applied to these embeddings as for the GO. We used GPT3.5 embeddings from scELMO (https://sites.google.com/yale.edu/scelmolib). The Random Forest Regressor model was tuned for the n_estimators hyperparameter. The Elastic Net model was tuned in a similar manner but for the l1_ratio hyperparameter, while the K-Nearest-Neighbors Regressor was tuned for the k neighbours'parameter. The best hyperparameter setting was selected based on the validation set performance in all models. The Random Forest Regressor, Elastic Net and k-Nearest-Neighbors were trained using the scikit-learn v1.5.2 Python package [32].

### Model evaluation

The model performance was evaluated using four metrics: 1) Pearson, 2) Pearson Delta, 3) Pearson Delta DE, and 4) Pearson Delta without target genes. The first three metrics were used in the scGPT paper. All metrics were calculated at the 'bulk' level, meaning that conditions (control and perturbed states) were mean aggregated over the gene dimension.

'Pearson' refers to the raw correlation between predicted and true post-perturbation expressions. 'Pearson Delta' refers to the correlation between the differential expression (post—control) of predicted and true post-perturbation expressions. 'Pearson Delta DE' is a variation of Pearson Delta that is calculated only for the top 20 most differentially expressed genes. 'Pearson Delta without target genes' excludes the CRISPR target gene(s) from the top 20 most differentially expressed genes.

### Gene embedding similarities

The biological coherence of the scGPT, scFoundation, scELMO, and GO gene embeddings was assessed by examining their correlation-based gene similarity networks in relation to well-characterized pathways from the Reactome [20] and KEGG [19] databases, as well as transcription factor-target interactions from the CollecTRI [21] database. To ensure consistency across comparisons, 16,239 genes common to all four embeddings were identified. For each embedding, gene vectors were extracted, and Pearson correlations were computed between all gene pairs within each pathway, generating a gene–gene correlation matrix. To evaluate the biological relevance of these embeddings, observed within-pathway correlations were compared against a null model in which gene vectors were randomly permuted, preventing any gene from retaining its original vector. For each pathway, the difference between real and randomized correlations was computed. The distributions of Pearson Delta values were visualized using bar plots, where each bar represented the mean Pearson delta across pathways with 95% confidence intervals.

## Supplementary Information

Supplementary Material 1: Supplementary Figure 1 – Subgroup analysis for Norman dataset Evaluation on the Norman dataset: Pearson delta metrics (y axis) for scGPT, scFoundation, Train Mean and Random Forest Regression with GO features (x axis, top) and for Random Forest Regression model with different features (x axis, bottom). Different subgroups (combo_seen0: none of the perturbations was present in the train set, combo_seen1: one of the perturbations was present in the train set, combo_seen2: both of the perturbations, but the combination was not seen in the train set, unseen_single: single perturbation, not seen in train set) are colour coded.

Supplementary Material 2: Supplementary Figure 2 – Gene embedding analysis Correlation between gene embeddings was calculated between genes corresponding to the same REACTOME (top left), KEGG (top right) pathways or CollecTRI (bottom) gene regulatory networks. The difference between gene embedding similarities and random gene pairs is shown (y, axis, pearson_delta, mean +/- 95% CI).

Supplementary Material 3: Supplementary Figure 3 – Norman and Replogle RPE1 data distribution Correlation heatmaps for pseudo-bulk differential expression signatures for Norman (left), and Replogle RPE1 (right) datasets. The black lines indicate the separation between training and test sets. Some samples (perturbations) are labelled on the x and y axes.

Supplementary Material 4: Supplementary Table 1: Number of different perturbations for benchmark datasets. Supplementary Table 2: Evaluation metrics for model benchmark analysis. Supplementary Table 3: Evaluation metrics for the subgroup analyisis of Norman dataset

## Data Availability

The code to reproduce our analysis is available at https://github.com/turbine-ai/PerturbSeqPredBenchmark.
