# Supplementary figures and images for: Benchmarking foundation cell models for post-perturbation RNA-seq prediction
Source: BMC Genomics. 2025 Apr 23;26:393. doi: 10.1186/s12864-025-11600-2 (PMC12016270; doi:10.1186/s12864-025-11600-2)

Supplementary Figure 1

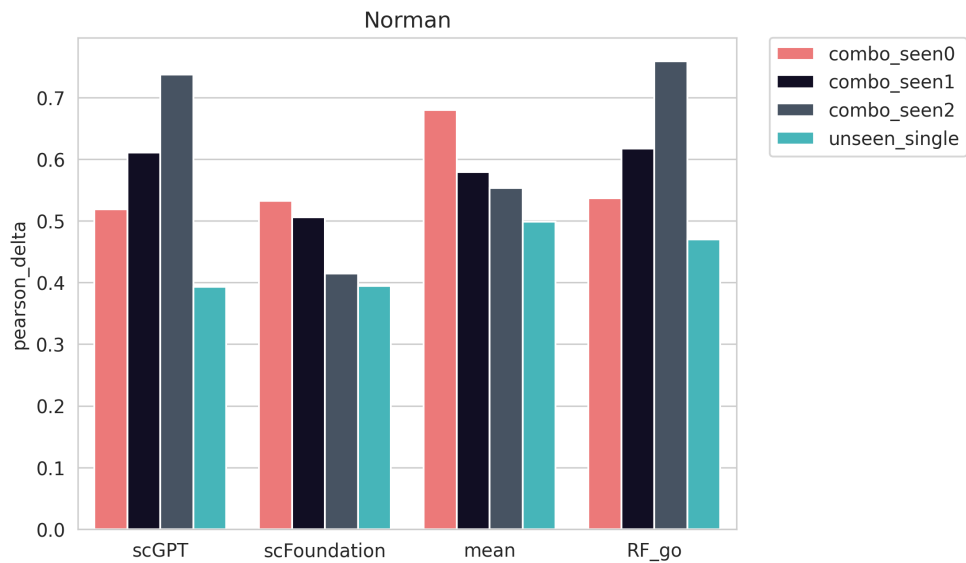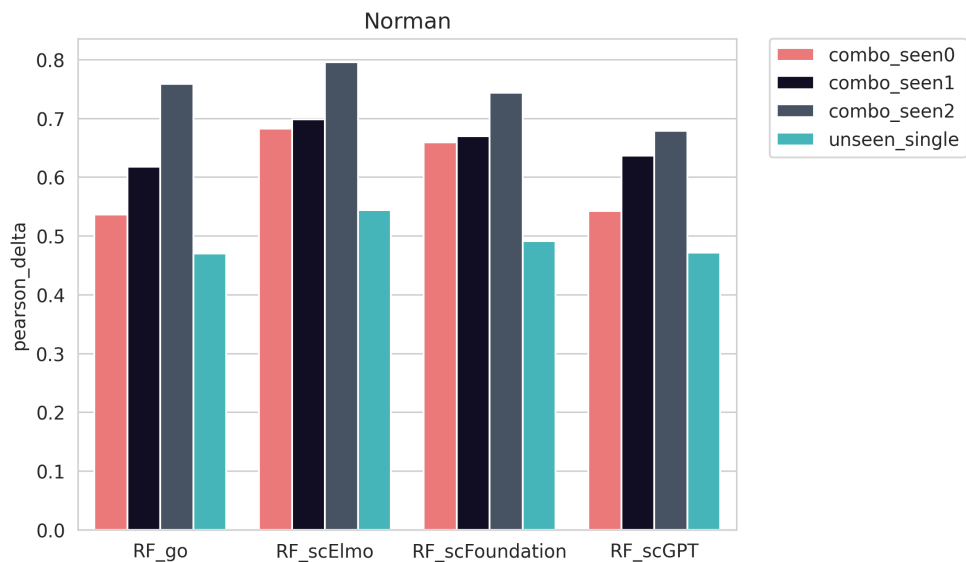

Supplement: Supplementary file 1 — Supplementary Material 1: Supplementary Figure 1 – Subgroup analysis for Norman dataset Evaluation on the Norman dataset: Pearson delta metrics (y axis) for scGPT, scFoundation, Train Mean and Random Forest Regression with GO features (x axis, top) and for Random Forest Regression model with different features (x axis, bottom). Different subgroups (combo_seen0: none of the perturbations was present in the train set, combo_seen1: one of the perturbations was present in the train set, combo_seen2: both of the perturbations, but the combination was not seen in the train set, unseen_single: single perturbation, not seen in train set) are colour coded. [file 12864_2025_11600_MOESM1_ESM.pdf]

Supplementary Figure 2

REACTOME

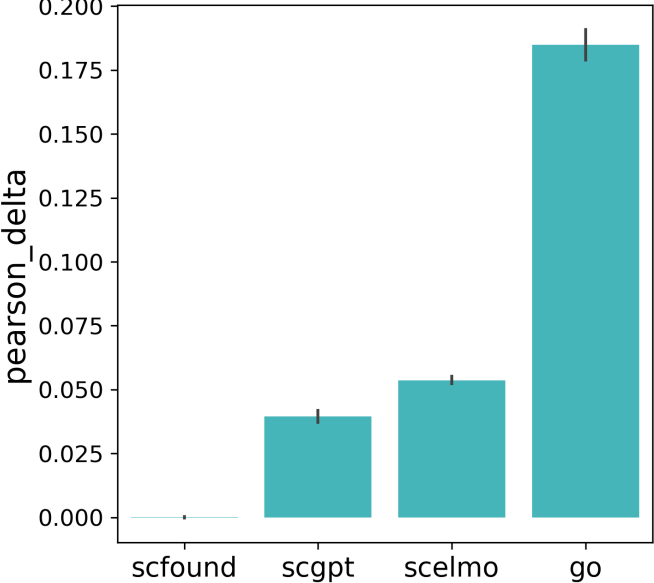

KEGG

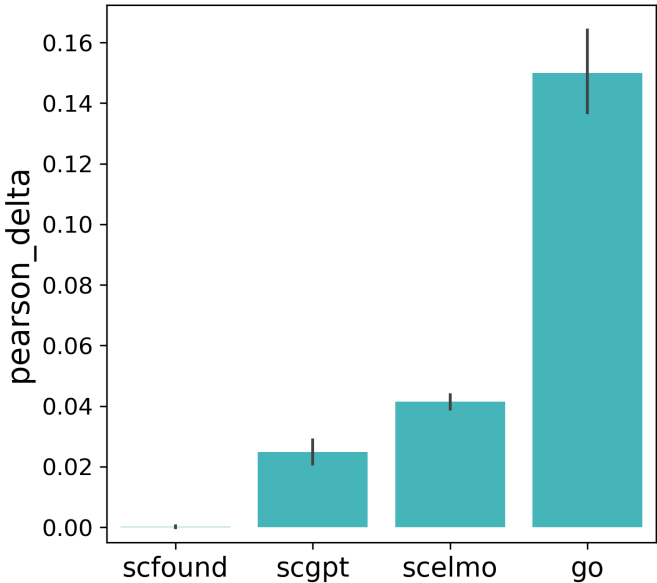

CollecTRI

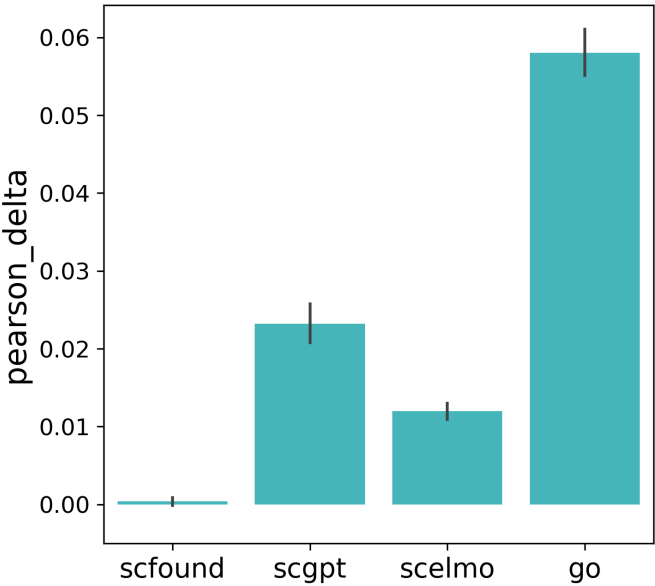

Supplement: Supplementary file 2 — Supplementary Material 2: Supplementary Figure 2 – Gene embedding analysis Correlation between gene embeddings was calculated between genes corresponding to the same REACTOME (top left), KEGG (top right) pathways or CollecTRI (bottom) gene regulatory networks. The difference between gene embedding similarities and random gene pairs is shown (y, axis, pearson_delta, mean +/- 95% CI). [file 12864_2025_11600_MOESM2_ESM.pdf]

# Supplementary Figure 3

**A**

**Norman**

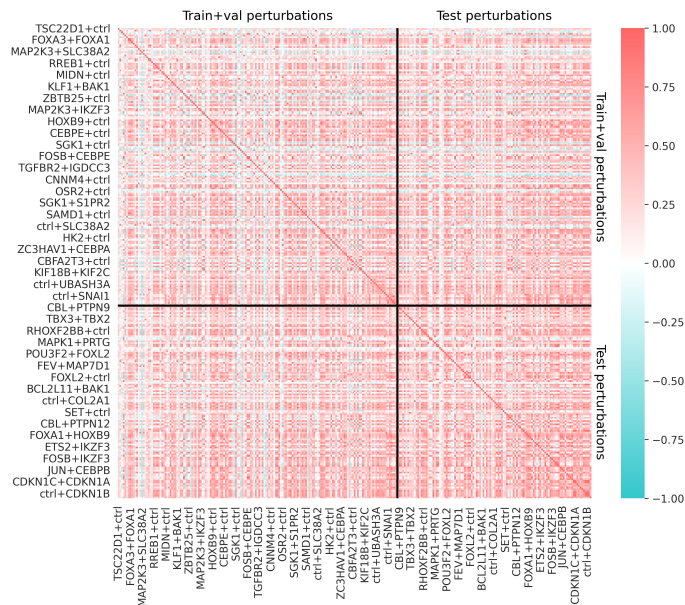

**B**

**Replogle RPE1**

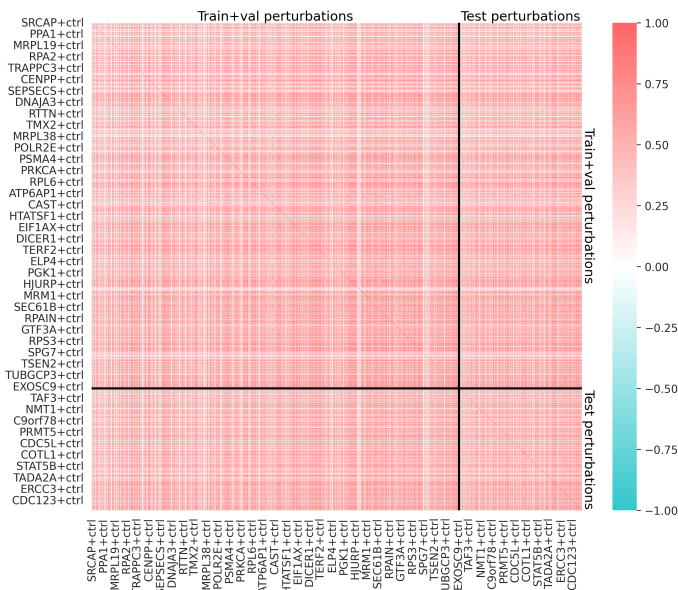

Supplement: Supplementary file 3 — Supplementary Material 3: Supplementary Figure 3 – Norman and Replogle RPE1 data distribution Correlation heatmaps for pseudo-bulk differential expression signatures for Norman (left), and Replogle RPE1 (right) datasets. The black lines indicate the separation between training and test sets. Some samples (perturbations) are labelled on the x and y axes. [file 12864_2025_11600_MOESM3_ESM.pdf]
